# Human breast cancer and sexual activities

**DOI:** 10.1038/sj.bjc.6604103

**Published:** 2008-01-22

**Authors:** N Akil, A Kassab, A Yasmeen, A D Darnel, T A Bismar, A-E Al Moustafa

**Affiliations:** 1 Faculty of Medicine/University of Aleppo, Aleppo, Syria; 2 Montréal Center for Experimental Therapeutics in Cancer, Lady Davis Institute for Medical Research of the Sir Mortimer B Davis-Jewish General Hospital, McGill University, Montreal, Quebec, Canada; 3 Program in Cancer Genetics, Department of Oncology, McGill University, Montreal, Quebec, Canada; 4 Department of Pathology Jewish General Hospital, McGill University, Montreal, Quebec, Canada

Sir,

Several studies have reported that high-risk human papillomaviruses (HPVs) are present in more than 50% of human breast cancers (Yu *et al*, 2000; Liu *et al*, 2001; Damin *et al*, 2004; de Villiers *et al*, 2005; Kan *et al*, 2005; Lawson *et al*, 2006a, 2006b; Yasmeen *et al*, 2007a, 2007b). Furthermore, high-risk HPVs of the same type were found in both cervical and breast cancer of the same patients (Hennig *et al*, 1999; Widschwendter *et al*, 2004). This finding has led to the hypothesis that HPVs could be transmitted to the breast through sexual activities (Kan *et al*, 2005; Lawson *et al*, 2006a, 2006b). Consequently, it is possible that the incidence of HPV-positive breast cancer in young women is related to high-risk HPV genital infections, which are much more common in young women who have multiple sexual partners (IARC, 1995).

There are three studies concerning the age of women in relation to the incidence of high-risk HPV-positive breast cancer. While there were no differences in the average age of women with either HPV-positive or -negative breast cancer in Brazilian women (Damin *et al*, 2004). In contrast, two recent studies reported that the average age of HPV-positive breast cancer in Greek and Australian women are 38 and 55.6 years in comparison with HPV-negative breast cancer, which are 53 and 63.8 years (*P*=0.001 and 0.049), respectively, (Kroupis *et al*, 2006; Lawson *et al*, 2006a, 2006b).

We recently reported that E6/E7 of HPV type 16 is present in the majority of invasive and metastatic breast cancer in comparison to normal mammary tissues in young Canadian women (Yasmeen *et al*, 2007a, 2007b). Moreover, we were able to demonstrate that the presence of E6/E7 of high-risk HPVs is correlated with invasive breast cancer in young Syrian women (Akil *et al*, in preparation); in this study, we documented the ages of Canadian and Syrian women in relation to their HPVs status. We found a statistically significant difference between the average age of women with high-risk HPV-positive breast cancers, which was 46.5 years in comparison with HPV-negative breast cancers of 57.5 years (*P*=0.05). Our data suggest that high-risk HPV-positive breast cancers are more frequent in younger women who are probably sexually more active than older women who have less sexual activity; thus, we firmly believe that high-risk HPV infections can be transmitted by sexual activity and could play an important role in the progression of human breast cancer. However, future epidemiological studies are necessary to confirm these findings.
